# 

**Published:** 1906-07-14

**Authors:** 


CONTENTS.

How Progress Came in English Medicine - - 257

Underfed Children - - - - - - - - - 258

Annotations - - - - - - - - - - - 259

Medical Opinion and Movement - - - - 260

Graduate Teaching Facilities in London Hospitals - - - - - - - - - 261

Practical Notes on Diagnosis and Treatment - 263

The Value of Anti-Malarial Public Works - - 263

Hospital Clinics—

A Clinical Lecture on Rheumatic Pericarditis - - - - 264

Special Analytical and Health Commission on Tinned and Preserved Food - - - - - 267

Remedies and their Uses - - - - - - - 268

The Book World of Medicine and Science - - 269

New Appliances and Things Medical - - - - 270

Medical Vacancies - - - - - - - - - 270

Hospital Administration—

Current Hospital Topics - - - - - - - 271

British and American Hospitals - - - - - - 272

Social and Poor-law Problems - - - - - - 273

Editor's Letter-Box - - - - - - - - 274

NURSING SECTION.

Notes on News from the Nursing World—
Registration and Parliament—The New Home at Newcastle—The Hop-picking Season—Colonial and British Nursing Homes Pupil Nurses in Fiji—Suggested Visiting Nurse for Huddersfield—Training at the National Hospital—Blackburn Operatives and Queen's Nurses—Amicable Settlement of a Libel Suit—The Outbreak of Typhoid at Belmont Asylum—Garden Party at Guy's Hospital—Nurses' Entertainment at St. Mary's Hospital—The Duchess of Marlborough and West Ham Hospital—Annual Reunion of Nottingham Nurses—St. Luke's Home for the Dying—Examination at Edmonton Infirmary—The Committee's Dilemma!—Progress at Lowestoft—District Nursing in Kingstown—Short Items - - - - - - 211-214
The Nursing Outlook - - - - - - - - 215
Abdominal Surgery - - - - - - - - - 216
The Nurse's Clinic - - - - - - - - - 217
Incidents in a Nurse's Life - - - - - - - 218
Home Preparations for Intending Probationers - 218
A Night with the Night Sister - - - - - 219
Association for Promoting the Training and Supply of Midwives - - - - - - - - 221
Everybody's Opinion - - - - - - - - 221
Appointments - - - - - - - - - - 222
"Cheer Up" Column - - - - - - - - 223
Presentations - - - - - - - - - - 223
The Nurses' Bookshelf - - - - - - - - 223
Notes and Queries - - - - - - - - - 224
For Reading to the Sick - - - - - - - 224

